# Endovascular repair of an ilio-iliac arteriovenous fistula following rupture of common iliac artery aneurysm with an aortic extension cuff in common iliac vein: A case report

**DOI:** 10.1097/MD.0000000000028548

**Published:** 2022-01-14

**Authors:** Young Sun Yoo

**Affiliations:** Department of Surgery, College of Medicine, Chosun University, Gwangju, South Korea.

**Keywords:** aneurysm, arteriovenous fistula, common iliac artery, endovascular repair

## Abstract

**Rationale:**

Common iliac artery aneurysm (CIAA) is complicated by ilio-iliac arteriovenous fistulas (IIAVF), which is rare but fatal and require prompt diagnosis and appropriate treatment. As open repair is associated with high morbidity and mortality, endovascular therapy is considered appropriate for treating an IIAVF.

**Patient concerns:**

A 76-year-old male patient who developed an IIAVF as a complication of ruptured CIAA, requiring immediate surgical repair presented to the hospital with hemodynamic instability.

**Diagnosis:**

Computed tomography angiography and conventional angiography revealed an IIAVF.

**Intervention:**

Endovascular therapy was selected to reduce the risk of morbidity and mortality. As the angiogram after the first endovascular aneurysm repair with stent-grafting showed contrast medium filling in the aneurysm sac, right common iliac vein, and the inferior vena cava, an aortic extension cuff was inserted into the right common iliac vein to close the orifice on the venous side.

**Outcomes:**

The right lower leg edema and discomfort were resolved immediately after the procedure, with the vital signs remaining stable. Computed tomography performed 6 months postoperatively showed patent stent-grafts of the artery and vein, with no evidence of IIAVF and endoleak.

**Lessons:**

IIAVF following CIAA rupture can be repaired successfully by stenting of the common iliac vein with an aortic extension cuff. For successful endovascular repair, the vein side of the fistula tract should be excluded with a stent-graft to block the backflow into the aneurysm sac.

## 1 Introduction

Ilio-iliac arteriovenous fistula (IIAVF), an arteriovenous fistula (AVF) between the common iliac artery (CIA) and common iliac vein (CIV), is an extremely rare condition that may occur due to trauma, iatrogenic injury, and aneurysm rupture.^[1,2]^ Particularly, IIAVF is a fatal complication of a ruptured common iliac artery aneurysm (CIAA) occurring in less than 1% of all CIAA cases.^[3,4]^ CIAA cases caused by trauma or iatrogenic injury are relatively quickly detected and treated; however, the clinical symptoms of IIAVF following an aneurysm appear only after its enlargement or rupture, which necessitates fistulous communication and introduces treatment difficulties.^[5,6]^

The presentation of IIAF may vary depending on its size and location; symptoms including abdominal pain, bruits and thrills, lower leg edema, and unilateral leg ischemia occur because of the changes in the artery-to-vein flow due to venous hypertension. This may ultimately induce a high cardiac output and, subsequently, cause heart failure.^[7]^ Therefore, in such cases, pulmonary embolism may occur because of distal embolization of the thrombus or atheroma in the aneurysm sac. Even anuria or hematuria may occur because of the functional renal damage due to venous congestion.^[5]^ If the condition worsens due to hemodynamic instability or multiple organ failure, emergency surgical intervention is required.^[8]^

Although early diagnosis and appropriate treatment are crucial to attaining good outcomes and preventing exacerbation of the condition, clinical practice guidelines for effective treatment remain unestablished as IIAF is extremely rare with limited relevant published data. Traditionally, surgical intervention has been the primary treatment choice, but less invasive endovascular treatment is performed when anatomically or technically suitable. Herein, we report the case of an IIAVF following CIAA rupture treated successfully by endovascular aneurysm repair (EAVR) using an aortic extension cuff on the CIV.

## 2 Case

**Figure 1:**
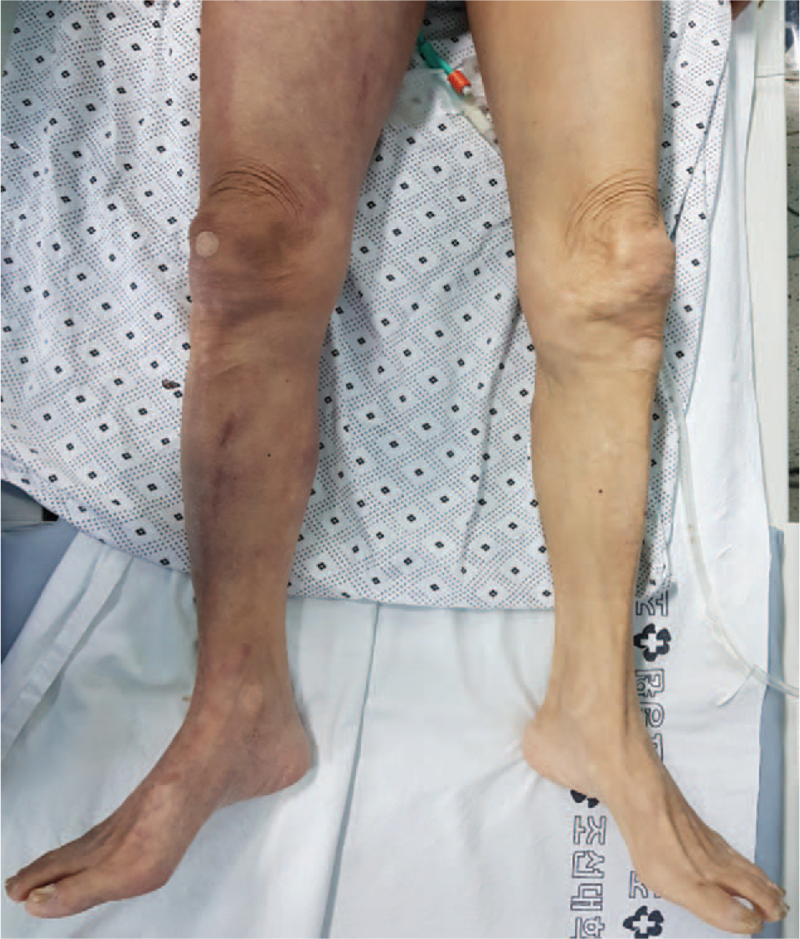
Clinical presentation at admission, showing edema and mottled skin color change on the right leg.

A 76-year-old male patient presented to the emergency department (ED) with a sudden onset of right lower leg edema and mottled skin color change that started 8 hours prior (Fig. 1). At admission, the patient had a blood pressure of 80/50 mm Hg, and a pulsatile mass in the right iliac fossa and thrill above the right groin was found on physical examination. The patient had hypertension and hyperlipidemia, with no history of trauma or open surgery, and he had no family history of abdominal aortic aneurysm. No other underlying conditions were present.

Laboratory workup revealed a hemoglobin level of 11.4 g/dL; creatinine level, 1.40 mL/dL (normal range: 0.5–1.3 mg dL); and normal electrolytes, liver enzymes, and acid-base balance. Troponin-T was slightly elevated to 0.036 (normal range: 0–0.0295), but no changes were observed on the electrocardiogram. The N-terminal prohormone of brain natriuretic peptide (NT-proBNP) was elevated to 686 pg/mL (normal range: 0–278 pg/mL).

**Figure 2:**
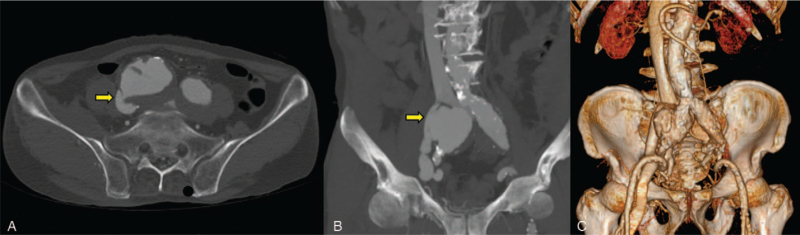
Contrast-enhanced computed tomography showing an isolated right common iliac artery aneurysm with evidence of an ilio-iliac arteriovenous fistula (arrow) in axial (A), sagittal (B), and 3D reconstruction (C) views. The contrast medium within right common iliac vein and inferior vena cava is visible.

Computed tomography (CT) angiography showed right CIAA measuring 54 mm in maximal diameter and an early filling of the contrast medium into the right CIV and inferior vena cava (IVC) in the arterial phase (Fig. 2). The aneurysm was ruptured, and an IIAVF had formed between the right CIA and right CIV. As the patient was experiencing rapid exacerbation with blood pressure falling to 60/40 mmHg with hemodynamic instability and intensified lower leg discomfort, emergency EVAR was scheduled.

Under general anesthesia, with a percutaneous approach using a suture-mediated closure system (Perclose ProGlide, Abbott Cardiovascular, Redwood, CA, USA) we performed a pre-close technique on both femoral arteries. Embolization of the right internal iliac artery (IIA) was attempted but it failed due to the fast blood inflow from the right CIA to the right CIV and a rigid stenosis at the opening. Hence, EVAR followed by surgical right IIA ligation was decided to be performed if any type II endoleak was identified. The stent-graft deployment was started: an aortic stent-graft system (Endurant IIs, Medtronic, Inc., Minneapolis, MN, USA) (25–14 mm × 103 mm) was inserted and a left iliac limb stent-graft (16–24/124 mm) was deployed. Because right IIA embolization was not performed, we deployed 2 iliac limb stent-grafts (16–16/93 mm, 16–10/124 mm) extending up to the right external iliac artery.

**Figure 3:**
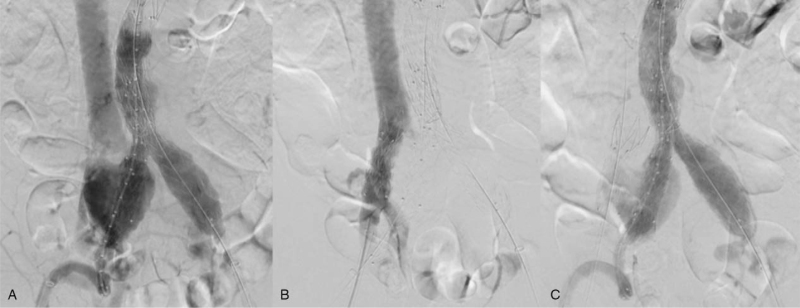
(A) Even after the stent-graft placement, the ilio-iliac arteriovenous fistula was visible. (B) The angiogram obtained after the deployment of aortic extension cuff in the right CIV showed no more contrast leakage in the venous part. (C) The angiogram obtained after the CIV stenting was completed showed relatively low leakage of contrast medium from the aneurysm sac, which was ascribed as remnant type-II endoleak from right internal iliac artery.

An angiogram obtained after stent graft insertion still showed the contrast medium filling in the aneurysm sac, right CIV, and IVC (Fig. 3A). Thus, we inserted an aortic extension cuff (23–23/49 mm) into the right CIV to close the venous orifice of the AVF. Contrast filling into the fistulous tract or aneurysm sac was not observed in the following angiogram of the venous side (Fig. 3B). A final angiogram was obtained after completion of the EVAR, showing contrast filling in the aneurysm sac, which was suspected as a type II endoleak from the IIA (Fig. 3C). Therefore, the patient was placed under observation. We investigated whether there was any direct blood inflow from the CIA to the CIV and IVC, suggestive of IIAVF; however, no such findings were observed.

**Figure 4:**
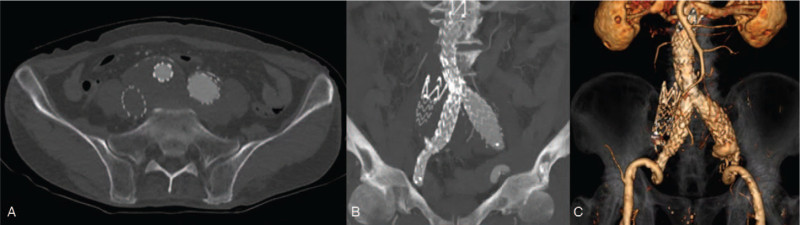
The follow up contrast-enhanced computed tomography at 6 months showed complete resolution of the arteriovenous fistula with no contrast leakage into aneurysm sac and good patency of stent-graft in the axial (A), sagittal (B), and 3D reconstruction (C) views.

The right lower leg edema and discomfort were resolved immediately after the procedure, with the vital signs remaining stable. The patient recovered without complications and was discharged on day 7 of hospitalization. CT performed 6 months postoperatively showed patent stent-grafts of the artery and vein, with no evidence of IIAVF and endoleak (Fig. 4). Currently, the patient has been maintained under outpatient follow-up for 18 months since the procedure. Informed consent was obtained from the patient for the publication of the case report and the accompanying image.

## 3 Discussion

As an IIAVF occurs rarely after CIAA, selecting its appropriate treatment strategy is difficult, especially since the clinical presentations widely vary and could be life-threatening. Furthermore, the treatment differs depending on anatomy, pathology, and availability of treatment options at each healthcare institution.

Open repair using a prosthetic graft was regarded as the gold standard.^[6,9,10]^ However, the critical aspect of open repair is the bleeding control strategy in the venous orifice. Huang et al introduced the method of controlling bleeding in an aneurysm sac by digital compression or balloon catheter insertion through the fistula tract for inflation and direct closure of the suture.^[4]^ However, failure to achieve proper proximal and distal control after opening the aneurysm sac causes heavy bleeding, which hinders direct suture repair. To control the bleeding on the venous side of the IIAF, extensive dissection up to the iliac vein is necessary to expose the proximal and distal aspects, which may worsen the risk of hemorrhages/hematomas. Moreover, complete hemostasis cannot be achieved through balloon catheter inflation, and calcification of the aneurysm wall hinders suturing. Eventually, conventional open repair increases the morbidity and mortality as the operation time and bleeding risk increases, which prolongs the postoperative recovery period. As reported, aneurysm rupture leads to a high mortality rate of 52%.^[2,3]^

With advances in endovascular techniques, less invasive endovascular repair has been performed when anatomically and technically suitable.^[8,11,12]^ This complicated condition is considered difficult to treat by conventional EVAR since it cannot block the blood flow from the vein to the aneurysm sac. The alternative solution to this is by graft stenting in the vein, but this can only be considered in traumatic or iatrogenic injury-related cases.^[13–15]^ In aneurysm-associated IIAVF cases, backflow occurs from the vein of the IIAVF into the aneurysm sac even after blocking the blood flow with a stent-graft, which may cause sac enlargement or delayed rupture. Thus, theoretically, a type II endoleak occurs and stent-grafts must be placed on both sides around the iliac artery and vein defects.

Rishi et al introduced the venous stent-graft placement strategy to prevent sac pressurization in IIAVF caused by CIAA rupture.^[8]^ They performed emergency EVAR without vein stenting in their patient who developed an IIAVF due to CIAA rupture. The CT from when the patient experienced a rapid exacerbation following their no-stent EVAR showed type IIIa endoleak caused by component separation. In that report, the continuous blood flow into the aneurysm sac through the fistula tract was attributed as the contributor of component separation. Furthermore, the patient experienced complete resolution of symptoms after relining of the arterial site and venous stent-grafting. Thus, theoretically, no hemodynamic changes were expected within the aneurysm sac due to the low pressure on the venous side. Nevertheless, venous stent-grafting should be performed to completely occlude the fistula orifice if possible. It must be noted that cases with long-term follow-up after vein stenting have not been reported.

The diameter and length of the available stent-grafts are factors that need consideration. The recommended diameter of iliac stents is 14 to 16 mm for general cases of stenosis and occluded lesions. However, an AVF distends the diameter of the iliac vein due to high flow; thus, the exact diameter of a suitable vein stent cannot be easily identified. While preoperative CT is useful as a reference, measuring the iliac vein diameter in the intraoperative angiogram may help. Because the vein distends further after resuscitation in cases of intraoperative hemodynamic instability, oversizing should be considered in such cases.

Accordingly, iliac extension limbs or aortic extension cuffs, or thoracic stent-grafts can be used, after selecting the diameter or length of the available stent-graft, especially in emergencies. Sueyoshi et al reported a case involving a long stent limb placement (16–20/124 mm) into the venous side because the exact AVF site could not be located during EVAR.^[12]^ They reported that the venous site was excluded, but the stent-graft was occluded due to the pressure from the iliac aneurysm. Therefore, they recommended the use of short stent-grafts with radial force on the vein side. We used an aortic extension cuff (49-mm length, 23-mm diameter) that covered a short range of the CIV and was suitable for the patient's venous diameter. This was possible because the AVF site had been accurately identified on CT and angiography. Thereafter, we could fit the CIV length accurately and oversized appropriately, resulting in an adequate radial force, which was vital for maintaining the patency of vein stenting. Thus, surgeons should use various types of stent grafts considering the CIV diameter or range of the AVF site.

One factor that reduces the flow into an aneurysm sac is occluding the venous blood flow and backflow from the internal iliac artery. Usually, IIA embolization is first attempted, but once an IIAVF is formed, the large flow volume from the iliac artery to the iliac vein causes contrast filling of these vessels on the angiogram, which hinders the identification of the IIA orifice. This occurs more frequently in cases involving IIA stenosis. Thus, when the patient is hemodynamically unstable, a stent-graft should be implanted from the aorta to the external iliac artery at the AVF site with venous stenting to occlude the fistulous channel followed by surgical IIA ligation if needed.

## 4 Conclusions

CIAA complicated by IIAVF can be successfully treated after judging the anatomical and pathological condition of the patient, as well as the available infrastructure at healthcare institutions. While there are many challenges in the treatment of such IIAVF cases because of the varied clinical presentations, endovascular treatment can be appropriately planned since it is less invasive and effective. Successful endovascular treatment of IIAVF following CIAA rupture is dependent on controlling the bleeding on the venous side and, if anatomically suitable, excluding the vein side using an aortic extension cuff is one viable treatment option.

## Acknowledgments

I would like to thank Editage (www.editage.co.kr) for English language editing.

## Author contributions

**Data curation:** Young Sun Yoo.

**Investigation:** Young Sun Yoo.

**Methodology:** Young Sun Yoo.

**Visualization:** Young Sun Yoo.

**Writing – original draft:** Young Sun Yoo.

**Writing – review & editing:** Young Sun Yoo.
